# Insights Into Type I and III Interferons in Asthma and Exacerbations

**DOI:** 10.3389/fimmu.2020.574027

**Published:** 2020-09-25

**Authors:** Helen E. Rich, Danielle Antos, Natalie R. Melton, John F. Alcorn, Michelle L. Manni

**Affiliations:** Department of Pediatrics, UPMC Children’s Hospital of Pittsburgh, Pittsburgh, PA, United States

**Keywords:** asthma, type I interferon, type III interferon, infection, interferon-alpha, interferon-beta, interferon-lambda, asthma therapeutics

## Abstract

Asthma is a highly prevalent, chronic respiratory disease that impacts millions of people worldwide and causes thousands of deaths every year. Asthmatics display different phenotypes with distinct genetic components, environmental causes, and immunopathologic signatures, and are broadly characterized into type 2-high or type 2-low (non-type 2) endotypes by linking clinical characteristics, steroid responsiveness, and molecular pathways. Regardless of asthma severity and adequate disease management, patients may experience acute exacerbations of symptoms and a loss of disease control, often triggered by respiratory infections. The interferon (IFN) family represents a group of cytokines that play a central role in the protection against and exacerbation of various infections and pathologies, including asthma. Type I and III IFNs in particular play an indispensable role in the host immune system to fight off pathogens, which seems to be altered in both pediatric and adult asthmatics. Impaired IFN production leaves asthmatics susceptible to infection and with uncontrolled type 2 immunity, promotes airway hyperresponsiveness (AHR), and inflammation which can lead to asthma exacerbations. However, IFN deficiency is not observed in all asthmatics, and alterations in IFN expression may be independent of type 2 immunity. In this review, we discuss the link between type I and III IFNs and asthma both in general and in specific contexts, including during viral infection, co-infection, and bacterial/fungal infection. We also highlight several studies which examine the potential role for type I and III IFNs as asthma-related therapies.

## Introduction

Asthma is a common chronic respiratory disease that affects approximately 300 million people worldwide and places significant economic burden on society. Asthma accounts for millions of disability-associated life years lost and over 200,000 deaths. In the United States between 2011 and 2016, 6.8% of working adults had asthma (11 million people) and nearly half reported an asthma exacerbation, with 10% having visited the emergency department over a 5 year span (1). In 2009, it was estimated that asthma was the cause of nearly 500,000 hospitalizations with an average stay of over 4 days, resulting in health care costs of 20 billion dollars (2). In children, asthma is the leading cause of chronic lung disease. Using the 2001–2016 National Health Interview Survey, asthma incidence in the United States was 9.2% in boys versus 7.4% in girls under the age of 18, with incidence increasing after 5 years of age (3). Further, asthma incidence and disease control also vary based on socioeconomic, genetic, and environmental factors. Children from low-income families, non-Hispanic Black children, and Puerto Rican children have higher incidence and reduced asthma control (4, 5). In 2013, 49% of asthmatic children missed school, 16.7% required an emergency department of urgent care visit, and 4.7% were hospitalized. This asthma burden resulted in over 13 million school days missed in the United States in a single year (2). Emergency department visits from exacerbations or acute attacks of asthma nearly double healthcare costs when compared with stable asthmatics (2). Despite advances in treatments, a significant portion of patients fail to achieve asthma control (6).

Asthma is a heterogeneous disorder characterized by airway inflammation, mucus hypersecretion, and partially reversible bronchial hyperresponsiveness with or without the presence of atopy and elevated immunoglobulin E (IgE). This complex respiratory disease encompasses a broad spectrum of phenotypes ranging from mild to severe disease, with varying degrees of responsiveness to steroid therapies. Based on lung function, medication use, and frequency of exacerbations, asthma is broadly defined as mild, moderate, or severe, and clinical characteristics are used to cluster adult and pediatric asthmatics (7–9). Although the majority of asthmatics have mild to moderate disease that is well managed with standard therapies, approximately 5–10% of asthmatics have severe disease, which comprises nearly 50% of the asthma-related healthcare costs (10, 11). To date, the presence and degree of type 2 inflammatory responses, involving eosinophilia and increased levels of the proinflammatory cytokines IL-4, IL-5, and IL-13, have been the focus of asthma research. Although the development of biologics that target pathologic type 2 inflammation have been successful in patients with disease marked by high eosinophilia (12, 13), approximately 50% of asthmatics do not exhibit this type 2 phenotype, especially those with severe corticosteroid refractory disease (14–16). Further, much less is known about pathogenic mechanisms in non-type 2 asthma. Clinical symptoms and steroid responsiveness have defined this subset of patients, but the need for more mechanistic studies focused on linking molecular mechanisms with clinical disease phenotypes is well appreciated.

Respiratory syncytial virus (RSV), human metapneumovirus (hMPV), rhinovirus (RV), and human parainfluenza virus (hPIV) represent four of the leading causes of respiratory tract infections in children and can lead to chronic wheezing and other pulmonary complications (17, 18). Numerous studies have linked childhood RV infection with wheeze (2, 19, 20). In infants, RSV is the most common cause of acute bronchiolitis and wheeze. Early life infection with RSV has been linked to type 2 immune activation and allergic sensitization (21). In addition to anti-viral inflammatory responses, viral infections also impact the microbiome. Bacterial outgrowth of *Moraxella catarrhalis*, *Haemophilus influenzae*, and *Streptococcus pneumoniae* has also been associated with wheeze (22). Despite these associations, the cause of asthma is still unknown, and many genetic and environmental factors are linked to the development of this chronic disease.

Exacerbations of asthma are acute or sub-acute episodes of worsening asthma symptoms and lung function. Asthma exacerbations account for the majority of the morbidity and mortality associated with this disease, health care costs, and loss of disease control (23, 24). Asthma exacerbations can be triggered by many factors, including but not limited to allergens, air and traffic pollution, upper and lower respiratory infections, cigarette smoking or vaping, and second-hand smoke or aerosol exposure (25, 26). It is well established that viral respiratory tract infections initiate the majority of exacerbations in both school-aged children and adults with asthma. Indeed, it is estimated that greater than 80% of asthma exacerbations are associated with viral infections (27). Many viruses have been identified as triggers of exacerbations including RV, RSV, hMPV, hPIV, influenza virus, coronavirus, enterovirus, bocavirus, and adenovirus (28). Human RV is commonly associated with asthma exacerbations and is detected in 76% of wheezing children and 83% of adult exacerbations (29, 30). Studies have shown that individuals with chronic airway diseases, like asthma, or chronic obstructive pulmonary disease (COPD), have impaired immune responses to infections, consequently triggering acute exacerbations of diseases. Recent research suggests that infants with deficient type I and III interferon (IFN) responses are more at risk for lower respiratory tract infections and wheezing later in their lives (31). As asthma exacerbations are commonly triggered by respiratory infections and type I and III IFNs are essential for antiviral host responses, we will review some common initiators of asthma exacerbations and type I and III IFN responses in the context of asthma and acute exacerbations. Finally, we will discuss several preventative measures and treatments that are utilized in preclinical and clinical settings.

## Type I and III Interferon Responses in the Lung

While type I IFNs have been known since 1957 as cell-secreted antiviral factors (32), and were the first cytokines discovered, type III IFNs (IFNλ, IL-28/29) were only first described in 2003. Their simultaneous discovery by two different groups led to their many names, with Paul Sheppard’s group calling them interleukins (IL)-29 and IL-28A/B (33), while Sergei Kotenko’s group referred to them as IFN lambda (IFNλ1/2/3, respectively) (34). While IFNλ1 is only found in humans, both mice and humans express IFNλ2 and IFNλ3. Though structurally dissimilar, type I and III IFNs converge at the beginning of their signal cascades to induce the transcription of a highly overlapping complement of interferon-stimulated genes (ISGs). However, the localization of the type III interferon-specific receptor IFNλR1 to mucosal tissues and immune cells restricts its actions (35). Type I and III IFNs also differ in their kinetics and ability to activate STAT1, leading to differences in IFN response factor expression and subsequent induction of pro-inflammatory chemokines (36). Moreover, more recent work shows that these differences may be independent of receptor abundance and instead intrinsic to their signaling pathways (37). While new research will continue to reveal differences between type I and III IFN signaling, these pathways have many redundancies and are highly overlapping throughout the respiratory tract (38, 39).

Interferon induction is perhaps best characterized in response to influenza infection in the lungs. Mice lacking the receptors for either type I (IFNαR1) or type III IFNs (IFNλR1) are more susceptible to influenza infection, and both are important for limiting mortality (40, 41). However, IFNαR1 deletion alone did not increase immunopathology in the lungs post influenza infection, suggesting that type III IFNs have an active anti-inflammatory role in this context. Further, type III IFNs are highly produced and less inflammatory than type I IFNs during influenza infection in the lungs (40). Type III IFNs did not induce the production of inflammatory cytokines in neutrophils and suppressed neutrophil migrations to sites of infection (40, 41), helping to limit pulmonary inflammation during influenza infection. While this reduction of neutrophils and resulting decrease in immunopathology is beneficial during influenza alone, neutrophils are necessary for antibacterial defense and thus the role of type I and III IFN responses may be different in the context of co-infection. During a co-infection, most commonly influenza and a secondary bacterial infection, both type I and III IFN are robustly produced after influenza infection and can be detrimental to host clearance of secondary bacterial infection (42, 43). Other models of co-infection exist, including RSV and *P. aeruginosa*. Biofilm growth of *P. aeruginosa*, a main factor for cystic fibrosis disease progression, was promoted by RSV infection and *P. aeruginosa* biofilm growth on polarized respiratory epithelium was enhanced by both type I and III IFN production (44). Thus, the anti-inflammatory effects of type III IFNs that are favorable to host outcomes during viral infection can limit the ability of the immune system to clear bacterial super-infection.

**FIGURE 1:**
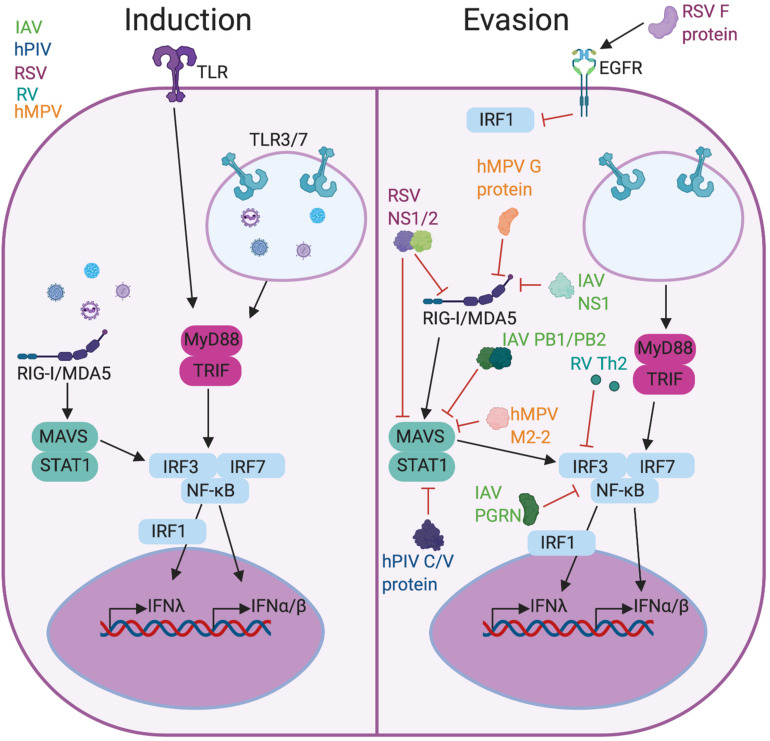
Viral induction and evasion of type I and III interferon response. Infection with RSV, RV, hPIV, hMPV, or IAV causes production of type I and III IFNs (left) through PRR signaling including TLRs and RLRs. These viruses have evolved functions to evade the IFN response (right) either by preventing PRR recognition or blocking the activity of downstream factors like IRF3 or STAT1. Figure created in BioRender.com.

In the lung, many viruses have mechanisms to impair or evade IFNs throughout the signaling pathway, affecting the ability of the immune system to recognize virus, control viral replication, and kill infected cells (Figure 1). RIG-I like receptors (RLRs) bind double-stranded RNA replication intermediates of these viruses and induce the production of type I and III IFNs. Initial detection of viral nucleic acids by RIG-I and MAVS is blocked by RSV proteins NS1/2, influenza A virus (IAV) NS1, PB1-F2/PB2, and hMPV G and M2-2 proteins (45–47). The hPIV V protein interacts with MDA5 to inhibit STAT activation and downstream signaling, and hPIV’s C and V proteins directly inhibit STAT1 phosphorylation in the IFNλ signaling cascade (48, 49). NF-κB and various interferon regulatory factors (IRFs), often IRF/3/7, are inhibited or degraded by IAV progranulin (PGRN) and type 2 cytokines produced by RV infection (50, 51). The RSV F protein also inhibits IRF1 outside of the classical IFNλ signaling pathway through activation of the epithelial growth factor receptor (EGFR) (52). Inhibition of IFN I and III can prolong infection in otherwise healthy patients and cause detrimental effects in compromised hosts, including asthmatics. As pathogens can play a role in asthma development or exacerbations, understanding the link between type I and III IFNs and asthma is crucial to combatting and controlling severe asthma.

## Type I and III IFNs and Asthma

In addition to controlling pulmonary infections, type I and III IFNs are also thought to regulate immune responses critical for asthma pathogenesis, but these mechanisms are less explored. While much research has focused on IFNγ as a pro-inflammatory mediator of severe asthma, altering airflow obstruction and steroid responsiveness (53, 54), type I and III IFNs have also been shown to be up-regulated in asthma. Children with asthma have increased expression of both IFNλ1 and IFNλ2 in their sputum, while adult asthmatics have increased sputum IFNλ2 but similar IFNλ1 levels when compared to healthy controls (55). Another study found elevated levels of IFN I and III in sputum of asthmatics with disease marked by neutrophilic inflammation (56). IFNα levels in sputum also correlated with higher levels of sputum lymphocytes in patients with asthma (57). In addition to type I and III IFNs, ISG activation is also prominent in mild and severe asthma, independent of viral transcripts and type 2 inflammation (58). Overall, type I and III IFN responses may influence asthma regardless of the degree of type 2 immune activation.

**FIGURE 2:**
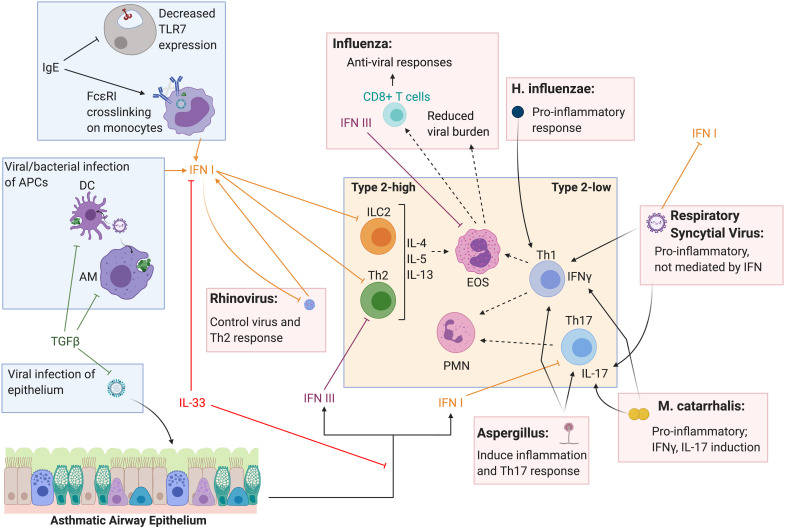
Type I and III interferon induction and T helper cell response to respiratory pathogens in the asthmatic lung. Type I and III IFNs are known to have overlapping innate and adaptive roles in the lung and decreased IFN response to respiratory infection in asthmatics is thought to contribute to acute exacerbations. Cross-inhibition between type 2 responses and type I and III IFNs have been reported in the context of type 2-high asthma and acute viral exacerbations. Type I and III IFNs also can alter Th1 and Th17 responses and may directly and indirectly influence type-2 low asthma and exacerbations of disease. Further, pathogens can influence T helper cell responses independent of IFN as well, directly altering the inflammatory environment in the asthmatic lung. Figure created in BioRender.com.

Evidence shows that type I and III IFNs can restrict the development of Th2 cells and secretion of type 2 cytokines, thereby mediating allergic responses (Figure 2). Type I IFNs have been shown to block Th2 development by suppressing GATA3 expression (59, 60) and altering Th2 cell activation and cytokine release (61–63). Similarly, the development and activation of human and murine Th17 cells are also negatively regulated by type I IFNs (64, 65). Further, recent work has also demonstrated a defect in type I IFN production in dendritic and epithelial cells from patients with severe atopic asthma (62, 66). Studies also show that type I IFNs are required for proliferation and effective transmigration of DCs in response to antigen and an optimal Th2 response *in vivo* (67–69). Using an ovalbumin murine model of asthma, all isoforms of type III IFNs were shown to alleviate allergic airway disease by reducing eosinophilia, decreasing type 2 cytokines, and modulating lung dendritic cell and CD4 + T cell functionality (70–72). Similarly, other studies have shown that IFNλ1 inhibits the development and responses of Th2 cells in human PBMCs in an IFNγ-dependent fashion (73, 74). Together, these studies suggest that type I and III IFNs regulate adaptive and innate immune cells that are critical to the development of allergic disease.

## Viruses in Asthma Exacerbations

It is well-appreciated that viruses are the cause of a significant portion of asthma exacerbations. In a cohort of 9–11 year old children studied over 13 months, 80–85% of asthma exacerbations occurred during viral infections (75). In one study, hPIV infection was found in 42% of asthma exacerbations, and children with hPIV-induced bronchiolitis can go on to develop chronic asthma due to virus-initiated immune reprogramming (76, 77). Similarly, over 50% of children with hMPV infections had wheezing complications and older children (5 and above) were likely to have asthma exacerbations due to hMPV (18, 78). Moreover, co-infection with multiple viruses can occur and increases the risk of asthma development. One study found that approximately 83% of children 6–8 years old with co-infection-induced bronchiolitis had recurrent wheezing as opposed to 70% of children with a single infection. The same study also found that hospitalizations due to co-infection were twice as high as single infection, indicating that co-infection is a higher risk factor for asthma exacerbation than single viral or bacterial infection (79).

Many studies have shown that host defense against respiratory viruses may be abnormal in patients with asthma. It has been speculated that asthmatics have a diminished capacity to overcome respiratory viruses due, in part, to low levels of IFNs in the bronchial mucosa. Several studies show that bronchial epithelial cells from pediatric and adult asthmatics have deficient induction of type I and III IFNs following RV infection (66, 80, 81), with the level of IFN production relating to the severity of infection (81, 82). Bronchial epithelial cells from asthmatics were shown to produce less type I and III IFN in response to viral challenge (21). Both IFNα and IFNβ were directly linked to more severe RV infection in a study that blocked type I IFN activity in healthy patients. Moreover, this study showed that otherwise healthy patients with impaired type I IFN mimicked what is seen naturally in asthmatics during infection (81, 83, 84). Mice with house dust mite (HDM)-induced allergic airway disease infected with influenza and primary bronchial epithelial cells from patients with mild, atopic asthma infected with RV produce IL-33 that subsequently suppresses production of type I IFNs (85). Interestingly, deficient immune responses to viral infection were not limited to patients with atopic, type 2-related disease, but were also present in those without type 2-associated conditions and severe therapy-resistant atopic asthma (66, 80, 86–88).

Several mechanisms for this apparent type 2 versus IFN cross-inhibition have been proposed in the context of asthma and acute viral exacerbations (Figure 2) (62). Reciprocally, type I IFN was shown to inhibit innate lymphoid cell 2 (ILC2) function as a mechanism of opposing type 2 inflammation (89). Further, moderate to severe asthmatics have been shown to express decreased levels of Toll-like receptor 7 (TLR7) on epithelial and innate immune cells, likely mediated by IgE, suggesting a defect in viral sensing and induction of IFNs (90, 91). Cross-linking of the IgE receptor, FcεRI, and increased FcεRI expression on plasmacytoid dendritic cells from atopic asthmatic children has been linked to decreased type I and III IFN production in response to RV (92) and influenza (93). Conversely, influenza infection in mice lacking the type I IFN receptor resulted in increased type 2 inflammation and IgE (89). A clinical trial using an IgE blocking antibody resulted in increased immune cell production of type I IFN upon *in vitro* stimulation with RV (94).

Airway inflammation in asthma is characterized by complex inflammatory protein interactions, and it is likely that more than one specific mediator or pathway influences and alters the lung environment. For instance, type I and III IFNs are known to have overlapping innate and adaptive roles as well as effects on other inflammatory mediators contributing to the complexity of understanding the mechanistic role of IFNs in this respiratory disease. The balance between asthma driving cytokines and those that render asthmatics more susceptible to viral infections and exacerbations is an important consideration when regarding IFNs as therapies.

## Influenza Infection in the Asthmatic Lung

The relationship between asthma and influenza is highly nuanced. Unlike other respiratory viruses, it has long been thought that asthmatics are no more likely than the general population to contract influenza. This has been contradicted by a study of the 2009 H1N1 pandemic which shows that children with asthma were twice as likely to be infected with H1N1 influenza compared with other respiratory viruses (95). However, infection of bronchial epithelium from human asthmatics and healthy controls with pandemic H1N1 influenza showed no difference in ability of virus to infect cells (96). Moreover, asthma did not increase H3N2 influenza viral shedding during *ex vivo* infection of bronchial biopsy explants when compared with those from healthy controls, both of which suggest that control of viral replication is maintained in the asthmatic lung (97). Importantly, a number of factors including RSV and RV co-infections during hospitalization for influenza may have complicated the analysis of the 2009 influenza pandemic (98), which may explain this discrepancy between experimental findings and epidemiological data. While there is a significant amount of data detailing the prevalence of asthma in people hospitalized for influenza, there is very little data concerning the incidence of influenza in asthmatics compared with healthy controls, making it impossible at this time to draw evidence-based conclusions regarding the effect of asthma on influenza susceptibility.

It has also been assumed for quite some time that asthmatics fare worse than the general population during influenza infection. Asthmatics were hospitalized earlier than non-asthmatics (99) during the 2009 H1N1 influenza pandemic but, surprisingly, were less likely to die (100). In a larger retrospective study of the pandemic outcomes, corticosteroid use and earlier hospital admission explained the lower death rate of asthmatics compared with healthy controls (101). In comparison, corticosteroid use has been shown to increase mortality from influenza in non-asthmatics (102). This pattern persisted across the world: a pooled global study of risk factors during the 2009 pandemic demonstrated that unlike all other chronic diseases assayed, asthma actually decreased the odds ratio for mortality compared with previously healthy people hospitalized for influenza (103).

This decrease in influenza mortality due to asthma is reproducible in rodent models. Mice with *Aspergillus fumigatus*-sensitized allergic airway disease (AAD) cleared H1N1 more rapidly than naïve mice (96). These results have been independently corroborated in a murine model of ovalbumin-sensitized AAD, where earlier clearance of H1N1 correlated with more rapid type III IFN induction in the ovalbumin-sensitized mice (104). It was observed that increased viral control correlated with higher numbers of eosinophils arriving earlier to the lung during influenza (96). More recent findings show that influenza exposure causes murine eosinophils to up-regulate the expression of genes encoding viral sensors (105), and that these eosinophils can become infected by influenza virus and degranulate in response to influenza (106). Strikingly, adoptive transfer of eosinophils into the airways of *A. fumigatus*-sensitized mice reduced influenza viral burden and weight loss in response to influenza infection, suggesting these cells are actively beneficial during influenza infection in mice with AAD. Moreover, this correlated with a higher number of virus-specific CD8 + T cells, and these influenza-exposed eosinophils were able to stimulate CD8 + T cell activation and proliferation *in vitro*, indicating a possible role for eosinophils as antigen-presenting cells in influenza infection during asthma (106). Eosinophils from human blood are also activated by influenza and are able to both uptake and inactivate fluorescent dye-labeled influenza virus. However, eosinophils from asthmatic patients were less able to capture influenza virus when compared with eosinophils from healthy controls, and this reduction correlated with severity of asthma (107). In summary, data from murine models suggest that eosinophils have direct antiviral activity and promote adaptive immunity against influenza during AAD. However, data from human eosinophils suggest their direct antiviral capacity may be reduced in asthma, leaving the possibility open that the reduction in influenza severity in asthmatics may be due to factors other than eosinophils.

While asthma appears to reduce severe outcomes from influenza infection, influenza can certainly exacerbate asthma. Influenza is often identified in sputum samples from asthma patients experiencing exacerbations (108), and children who did not receive the influenza vaccine were more likely to have asthma exacerbations (109). While there has been some concern in the lay community that influenza vaccination itself could cause acute asthma exacerbations, a study encompassing more than 1 million children in the United States over three influenza seasons from 1993–1996 showed no increase in asthma exacerbations in both a 2-day and 2-week period after vaccination (110). Importantly, children with more severe asthma are more likely to receive the influenza vaccine (111), creating a confounding variable. Without taking that confounder into account, analysis suggested that vaccinated children were more likely to experience exacerbations. However, upon controlling for asthma severity, the analysis revealed that children who had received the influenza vaccine were in fact less likely to have asthma exacerbations in the 2-week period following vaccination (110).

The molecular mechanisms by which influenza exacerbates asthma are still somewhat unclear. The decreased type I IFN response to viral infection in asthmatics may aid them during influenza infection, but it likely contributes to the aggravation of type 2 immunity during influenza-induced asthma exacerbations, as type I IFNs suppress type 2 immunity. In fact, type 2 cytokines, which dominate the most studied endotype of asthma, have been shown to be increased by influenza. In mice with HDM-sensitized AAD, influenza infection increased mucus production, pulmonary inflammation, and airway hyper-responsiveness (AHR), the hallmarks of asthma pathologies. Analysis of BAL and lungs showed much higher cellular inflammation in the influenza-infected, HDM-sensitized mice compared with mice that were only infected with influenza. This was correlated with early high IL-33 that persisted throughout influenza infection, and later induction of myriad pro-inflammatory mediators including KC, TNFα, IL-6, IL-12p40, IL-17A, CCL2, CCL20, and RANTES (112). This same group later showed a key role for IL-33 as a driver of asthma exacerbations: antibody blockade of the IL-33 receptor reduced AHR as effectively as systemic corticosteroids (85). While this group found no role for the IL-33-producing ILC2 cells in influenza exacerbation of asthma, another group using the same HDM-sensitized murine model implicated ILC2s as well as CD4 + T cells. While ILC2s were present earlier in the lung than T cells, their numbers did not increase due to influenza infection, and CD4 + T cells were able to produce pathogenic type 2 cytokines earlier during influenza-induced asthma exacerbation. Only during viral clearance, when ILC2 numbers in the BAL fluid were declining, did ILC2s produce a meaningful amount of type 2 cytokines (113). While the epidemiology unambiguously shows that influenza causes asthma exacerbations, the roles of specific cytokines and immune cells involved still merit significant study, especially the influence of type I and III IFNs that are so highly produced in healthy patients in response to influenza.

## Bacteria and Fungi in Asthma Exacerbations

While virus infections are thought to be the main culprit of infection-associated asthma exacerbations, they are not the only pathogens that contribute to exacerbations. Both bacteria and fungi that cause respiratory infections are associated with higher risk of exacerbation in asthmatics. Studies have shown that neonates colonized with *S. pneumoniae*, *M. catarrhalis*, or *H. influenzae* have increased risk of airway inflammation during infection and developing asthma later in life (114). Additionally, a longitudinal study showed that sensitization to *S. aureus* enterotoxins increased risk of severe asthma and asthma exacerbations up to 20 years after the study began (115).

In considering how asthma patients will respond to bacteria and fungi, type I and type III IFN again are important factors. For example, asthmatics have increased risk of severe *S. pneumoniae* infection compared to their healthy counterparts. Studies in mice have shown that prophylactic IFNα administration increases macrophage and neutrophil activation upon *S. pneumoniae* infection, leading to faster clearance of bacteria and reduced lung inflammation (116, 117). As asthmatics often have lower IFN responses to pathogens, this may impair their defenses against exacerbation-causing bacteria as well as viruses, underscoring the importance of developing IFN-based therapies.

The Gram-negative bacteria *M. catarrhalis* and *H. influenzae* have also been associated with wheezing. Colonization of the airways with either of these bacteria during childhood increased the likelihood of asthma diagnosis later in life (114). In one study, 21% of infants tested were colonized with *S. pneumoniae, M. catarrhalis, H. influenzae*, or a combination; of these infants, colonization with one or more of the above correlated with persistent wheezing along with elevated eosinophil counts and serum IgE levels (118). Additionally, infants dominated by *H. influenzae* had more instability in their microbiome over time, which led to more frequent respiratory infections compared to infants with a stable microbiome (119). Non-typeable *H. influenzae* (NTHi) induces a potent inflammatory response upon infection, including IL-8, TNFα, and IFNγ. IFNγ has been suggested as a therapeutic for recurrent NHTi infections but has not been sufficiently tested (120). Similarly, *M. catarrhalis* colonization can lead to asthma exacerbations through massive production of inflammatory mediators like IL-6, TNFα, IFNγ, and IL-17 (121). Therapies in the form of neutralizing antibodies against both IL-6 and TNFα have proven effective in mice against *M. catarrhalis*-caused asthma exacerbations, but IFNs have not been studied as *M. catarrhalis* efficiently down-regulates TLR3 in infected cells, resulting in almost complete ablation of IFNβ, IFNλ, and IL-8 secretion (122).

While bacteria can exacerbate asthma on their own, they are also found during viral-bacterial co-infections in the lung, which as previously discussed most often occurs during influenza infection. Like influenza, it appears that asthma may protect patients from severe disease during co-infection with influenza and bacteria. A murine model of ovalbumin-sensitized AAD showed that sensitized mice had increased bacterial clearance and survival after influenza/*S. pneumoniae* co-infection as compared to mice without AAD. Furthermore, these results were repeated with HDM-sensitized mice, which also displayed lower bacterial burden and mortality in response to influenza/*S. pneumoniae* co-infection compared with non-sensitized mice. The mice with AAD produced more TGFβ even before influenza infection, and this protection from infectious disease was ablated in mice with deletion of TGFβRII (123). TGFβ is commonly up-regulated in asthma (124), and is thus likely to contribute to protection from viral/bacterial co-infection in humans with asthma as well. An independent group corroborated these findings in a model of *A. fumigatus*-sensitized AAD, showing that bacterial burden and mortality were decreased during influenza/*S. pneumoniae* co-infection in sensitized mice compared with healthy controls (125). As type I and III IFNs are such important mediators of influenza-induced susceptibility to secondary bacterial infection, it is likely that they are altered by preceding asthma, but measurements of these IFNs were not reported in either study.

*Aspergillus fumigatus* infects both healthy and immunocompromised individuals, but even colonization without invasive infection in asthmatics can result in sensitization and AHR that increase the likelihood for an exacerbation (126). While *A. fumigatus* can be used to induce AAD in mice and contributes to the development of asthma in humans, it can also invade the lung causing invasive aspergillosis, as well as causing a number of pulmonary diseases (127). Both type I and type III IFNs are robustly induced upon infection with *A. fumigatus* and help the host to clear the fungus. Specifically, CCR2 + monocytes are primarily responsible for promoting type I IFN production upon *A. fumigatus* infection, and the presence of type I IFNs allows for optimal IFNλ signaling later in infection (128). Once IFNλ is produced with the help of type I IFNs, it acts directly on neutrophils to promote antifungal activity and clear the infection (128). The effectiveness of IFNs in clearing *A. fumigatus* infection makes them attractive therapeutic candidates. It has been postulated that the regulation of neutrophils and ROS by IFNλ could be used in a therapeutic setting, but more work needs to be done in this area (129). In summary, IFN I and III aid host defense against bacteria and fungi as well as viruses in the lung and make attractive targets for boosting immunity against this plethora of pathogens that contribute to asthma exacerbations. However, the research regarding IFNs as treatments is limited and will require further studies to evaluate their potential in these settings.

## Clinical Implications of Type I and III Interferon Therapies

Inhaled corticosteroids (ICS) are commonly prescribed therapies in airway diseases, such as COPD and asthma, and are used to improve disease control and reduce asthma exacerbations. However, this course of treatment may not be the ideal or efficacious solution for all patients, particularly those with more severe asthma, non-type 2 responses, or early in exacerbations when airway neutrophilia is high. Evidence also suggests that corticosteroids may impair innate antiviral immune responses and may contribute to increased risk of exacerbations and severity of disease. Indeed, McKeever and colleagues showed that asthmatics receiving ICS have an increased risk of pneumonia or lower respiratory infection, with those receiving higher doses being at greater risk (130). Further, suppression of IFNs by ICS during virus-induced COPD exacerbations mediated pneumonia risk, suggesting that inhaled IFNβ therapy may be protective (131). These studies suggest that suppression of IFNs by corticosteroids may render patients with preexisting airway disease more susceptible to viral infections and exacerbations, thus, type I and III IFN therapy may be beneficial in some settings. Outside of the lung, type I and III IFNs have been explored as treatments and therapeutic targets for a variety of inflammatory illnesses, including sepsis, cancer, ocular disease, and rheumatoid arthritis (132–135). It is therefore worthwhile to examine potential uses of type I and III IFNs within the lung as well.

As type I and III IFNs can restrict the secretion of Th2 cytokines and mediate allergic responses, the therapeutic potential of these IFNs for the treatment of asthma and asthma exacerbations has been explored. Indeed, intranasal administration of human IFNλ1 attenuated eosinophilic inflammation in the airways, production of IL-4, IL-5, and IL-13 in the lung, and pulmonary resistance in mice with ovalbumin-induced AAD (136). Similarly, asthmatic mice that received IFNλ2/3 intranasally exhibited significant decreases in TSLP and IL-33 protein levels in the BAL fluid, less lung inflammation by histology, and improved pulmonary resistance (71). Other groups have shown that treatment of human PBMCs with IFNλ1 inhibits the development and responses of Th2 cells, primarily by diminishing IL-13 secretion while not inducing a complementary elevation in IFNλ (73, 74). In addition to IFNλ1, other isoforms have also been studied for their therapeutic potential. Specifically, Koltsida and colleagues demonstrated that overexpression of IFNλ2 in the lung inhibited Th2 and Th17 responses and suppressed OVA-induced AAD in mice (72). Further, this IFN-induced suppression was dependent on IFNγ and IL-12 (72).

Beyond type III IFNs, studies outline the therapeutic potential of type I IFNs in asthma control. Several publications show that treatment with IFNα, coupled with corticosteroids, to be beneficial in poorly-controlled asthma, citing improved lung functionality and decreased AHR (137–139). Similarly, IFNβ was also shown to inhibit AHR in a murine model of asthma (140). In the context of exacerbations, a clinical trial of exogenous IFNβ treatment at the onset of cold symptoms improved peak expiratory flow and asthma control questionnaire score in severe asthmatics (141). When IFNβ was administered to asthmatic patients infected with RV, only slight improvements in morning peak expiratory flow recovery were observed (141). As the vast majority of this research has been focused in type 2 driven disease, it is still unclear if type I and III IFNs have a potential therapeutic role in severe, type 2-low driven disease. IFNγ has been identified as a driver of severe, steroid unresponsive asthma. Studies have shown IFNγ^+^ CD4^+^ T cells are more prevalent in the airways in severe asthma versus mild, moderate disease and that IFNγ-induced expression of CXCL10 and down-regulation of SLPI lead to increased AHR and steroid resistance in severe asthma (53, 54, 142, 143). Thus, the asthma endotype may need to be considered in the context of type I and III IFNs.

While type I and III IFNs have significant antiviral activity and are important in bacterial infection of the lung, evidence shows that they also have important immunoregulatory properties, especially in the lung. While the therapeutic applications of type I and III IFNs are still emerging, several preclinical and clinical studies show the effects of IFN treatments on pulmonary diseases (Table 1).

**TABLE 1:** Therapeutic Applications of Type I and III IFNs in Asthma (in chronological order).

References	Tested intervention/Drug	Subjects/Study population	Outcomes
**Preclinical:**			
Maeda et al. 1997 (140)	IFNβ via intraperitoneal administration and prednisolone treatments	Mice with type 2 dominant allergic airway disease	Improved lung inflammation and reduced AHR, with no change in secreted IgE
Li et al. 2014 (70)	Ad-hIFNλ1 via intranasal administration	Mice with type 2 dominant allergic airway disease	Improved lung inflammation (lower IL-4, IL-5, and IL-13) and decreased eosinophilia
Won et al. 2019 (71)	IFNλ2/3 via intranasal administration	Mice with type 2 dominant allergic airway disease	Improved lung inflammation (lower TSLP and IL-33)
**Clinical:**			
Gratzl et al. 2000 (137)	Administration of IFNα daily for ∼6 months	Case study of a 38-y/o with poorly controlled eosinophilic asthma	Reduced IL-5 release from PBMCs, decreased blood eosinophils, and possibly increased corticosteroid sensitivity
Simon et al. 2003 (138)	Treatment with IFNα over the course of 5–10 months	10 adults with severe steroid-resistant asthma taking prednisone	Improved lung function, lowered required dose of corticosteroids, decreased blood leukocytes, increased IL-10 expression in PBMCs, and promoted Th1 differentiation
Kroegel et al. 2009 (139)	Treatment with IFNα over the course of 12 months	16 adults with severe, persistent asthma on long-term oral glucocorticoid treatment	Improved lung function, lowered required dose of corticosteroids, decreased blood eosinophils, and decreased asthma-associated emergency room visits and hospitalizations
Djukanović et al. 2014 (141)	Inhaled administration of IFNβ daily for 14 days after onset of cold symptoms	Asthmatic patients on inhaled corticosteroids	Enhanced morning peak expiratory flow recovery, reduced need for treatment, and increased ISGs in sputum cells

## Summary

The importance and necessity of both type I and type III IFNs is universal in viral, bacterial, and fungal infections in the lungs. With infection being a prominent cause of asthma exacerbation in both children and adults, understanding the role of IFNs may be crucial to preventing and treating exacerbations. While the role of type II IFN (IFNγ) in asthma has been the subject of considerable investigation, new research shows that type I and III IFNs may also have a hand in asthma development and exacerbation. Here, we have discussed current knowledge regarding the role of type I and III IFNs in the development of asthma and in defense against common respiratory pathogens linked to asthma exacerbation. Finally, we summarize the current state of type I and III IFN-based therapies for asthma.

## Author Contributions

HR, DA, NM, JA, and MM performed literature searches, drafted, and critically revised this work. All authors contributed to the article and approved the submitted version.

## Conflict of Interest

The authors declare that the research was conducted in the absence of any commercial or financial relationships that could be construed as a potential conflict of interest.

## Abbreviations

AAD: allergic airway disease
AHR: airway hyperresponsiveness
EGFR: epithelial growth factor receptor
hMPV: human metapneumovirus
hPIV: human parainfluenza virus
IAV: influenza A virus
ICS: inhaled corticosteroids
IFN(s): interferon(s)
IgE: immunoglobulin E
IRF: interferon regulatory factor
MDA5: melanoma differentiation-associated protein 5
NTHi: non-typeable *Haemophilus influenza*
PBMCs: peripheral blood mononuclear cells
pDCs: plasmacytoid dendritic cells
PGRN: progranulin
RIG-I: retinoic acid-inducible gene I
RLR: RIG-I like receptor
ROS: reactive oxygen species
RSV: respiratory syncytial virus
RV: rhinovirus
TGF β: transforming growth factor beta
TLR: Toll-like receptor.

## References

[1] Mazurek JM Syamlal G. Prevalence of asthma, asthma attacks, and emergency department visits for asthma among working adults – National health interview survey, 2011–2016. *MMWR Morb Mortal Wkly Rep.* (2018) 67:377–86. 10.15585/mmwr.mm6713a1 29621204 PMC5889242

[2] Gern JE. How Rhinovirus infections cause exacerbations of asthma. *Clin Exp Allergy.* (2015) 45:32–42. 10.1111/cea.12428 25270551

[3] Zahran HS Bailey CM Damon SA Garbe PL Breysse PN. Vital signs: asthma in children – United States, 2001–2016. *MMWR Morb Mortal Wkly Rep.* (2018) 67:149–55. 10.15585/mmwr.mm6705e1 29420459 PMC5812476

[4] Akinbami LJ Moorman JE Bailey C Zahran HS King M Johnson CA Trends in asthma prevalence, health care use, and mortality in the United States, 2001-2010. *NCHS Data Brief.* (2012) 94:1–8. 22617340

[5] Barr RG Avilés-Santa L Davis SM Aldrich TK Gonzalez F Henderson AG Pulmonary disease and age at immigration among hispanics. results from the hispanic community health study/study of latinos. *Am J Respir Crit Care Med.* (2016) 193:386–95. 10.1164/rccm.201506-1211OC 26451874 PMC4803083

[6] Pavord ID Beasley R Agusti A Anderson GP Bel E Brusselle G After asthma: redefining airways diseases. *Lancet.* (2018) 391:350–400. 10.1016/S0140-6736(17)30879-6 28911920

[7] Moore WC Meyers DA Wenzel SE Teague WG Li H Li X Identification of asthma phenotypes using cluster analysis in the Severe Asthma Research Program. *Am J Respir Crit Care Med.* (2010) 181:315–23. 10.1164/rccm.200906-0896OC 19892860 PMC2822971

[8] Fitzpatrick AM Teague WG Meyers DA Peters SP Li X Li H Heterogeneity of severe asthma in childhood: confirmation by cluster analysis of children in the national institutes of health/national heart, lung, and blood institute severe asthma research program. *J Allergy Clin Immunol.* (2011) 127:382–9.e1–13. 10.1016/j.jaci.2010.11.015 21195471 PMC3060668

[9] Chung KF Wenzel SE Brozek JL Bush A Castro M Sterk PJ International ERS/ATS guidelines on definition, evaluation and treatment of severe asthma. *Eur Respir J.* (2014) 43:343–73. 10.1183/09031936.00202013 24337046

[10] Fitzpatrick AM Baena-Cagnani CE Bacharier LB. Severe asthma in childhood: recent advances in phenotyping and pathogenesis. *Curr Opin Allergy Clin Immunol.* (2012) 12:193–201. 10.1097/ACI.0b013e32835090ac 22249197 PMC3310912

[11] Sullivan SD Rasouliyan L Russo PA Kamath T Chipps BE Group TS. Extent, patterns, and burden of uncontrolled disease in severe or difficult-to-treat asthma. *Allergy.* (2007) 62:126–33. 10.1111/j.1398-9995.2006.01254.x 17298420

[12] Wenzel S Ford L Pearlman D Spector S Sher L Skobieranda F Dupilumab in persistent asthma with elevated eosinophil levels. *N Engl J Med.* (2013) 368:2455–66. 10.1056/NEJMoa1304048 23688323

[13] Ray A Raundhal M Oriss TB Ray P Wenzel SE. Current concepts of severe asthma. *J Clin Invest.* (2016) 126:2394–403. 10.1172/JCI84144 27367183 PMC4922699

[14] Fajt ML Wenzel SE. Asthma phenotypes and the use of biologic medications in asthma and allergic disease: the next steps toward personalized care. *J Allergy Clin Immunol.* (2015) 135:299–310; quiz 1. 10.1016/j.jaci.2014.12.1871 25662302

[15] Modena BD Tedrow JR Milosevic J Bleecker ER Meyers DA Wu W gene expression in relation to exhaled nitric oxide identifies novel asthma phenotypes with unique biomolecular pathways. *Am J Respir Crit Care Med.* (2014) 190:1363–72. 10.1164/rccm.201406-1099OC 25338189 PMC4294630

[16] Woodruff PG Modrek B Choy DF Jia G Abbas AR Ellwanger A T-helper type 2-driven inflammation defines major subphenotypes of asthma. *Am J Respir Crit Care Med.* (2009) 180:388–95. 10.1164/rccm.200903-0392OC 19483109 PMC2742757

[17] Coverstone AM Wang L Sumino K. Beyond respiratory syncytial virus and rhinovirus in the pathogenesis and exacerbation of asthma: the role of metapneumovirus, bocavirus and influenza virus. *Immunol Allergy Clin North Am.* (2019) 39:391–401. 10.1016/j.iac.2019.03.007 31284928 PMC7127190

[18] Rudd PA Thomas BJ Zaid A MacDonald M Kan-O K Rolph MS Role of human metapneumovirus and respiratory syncytial virus in asthma exacerbations: where are we now? *Clin Sci.* (2017) 131:1713–21. 10.1042/CS20160011 28667069

[19] Jartti T Gern JE. Role of viral infections in the development and exacerbation of asthma in children. *J Allergy Clin Immunol.* (2017) 140:895–906. 10.1016/j.jaci.2017.08.003 28987219 PMC7172811

[20] Jartti T Bønnelykke K Elenius V Feleszko W. Role of viruses in asthma. *Semin Immunopathol.* (2020) 42:61–74. 10.1007/s00281-020-00781-5 31989228 PMC7066101

[21] Edwards MR Strong K Cameron A Walton RP Jackson DJ Johnston SL. Viral infections in allergy and immunology: how allergic inflammation influences viral infections and illness. *J Allergy Clin Immunol.* (2017) 140:909–20. 10.1016/j.jaci.2017.07.025 28987220 PMC7173222

[22] Bisgaard H Hermansen MN Bønnelykke K Stokholm J Baty F Skytt NL Association of bacteria and viruses with wheezy episodes in young children: prospective birth cohort study. *BMJ.* (2010) 341:c4978. 10.1136/bmj.c4978 20921080 PMC2950260

[23] O’Byrne PM Pedersen S Lamm CJ Tan WC Busse WW Group SI. Severe exacerbations and decline in lung function in asthma. *Am J Respir Crit Care Med.* (2009) 179:19–24. 10.1164/rccm.200807-1126OC 18990678

[24] Ivanova JI Bergman R Birnbaum HG Colice GL Silverman RA McLaurin K. Effect of asthma exacerbations on health care costs among asthmatic patients with moderate and severe persistent asthma. *J Allergy Clin Immunol.* (2012) 129:1229–35. 10.1016/j.jaci.2012.01.039 22326484

[25] Murrison LB Brandt EB Myers JB Hershey GKK. Environmental exposures and mechanisms in allergy and asthma development. *J Clin Invest.* (2019) 129:1504–15. 10.1172/JCI124612 30741719 PMC6436881

[26] Chau-Etchepare F Hoerger JL Kuhn BT Zeki AA Haczku A Louie S Viruses and non-allergen environmental triggers in asthma. *J Investig Med.* (2019) 67:1029–41. 10.1136/jim-2019-001000 31352362 PMC7428149

[27] Ritchie AI Farne HA Singanayagam A Jackson DJ Mallia P Johnston SL. Pathogenesis of viral infection in exacerbations of airway disease. *Ann Am Thorac Soc.* (2015) 12 (Suppl. 2):S115–32. 10.1513/AnnalsATS.201503-151AW 26595727

[28] Fokkens WJ Garcia-Garcia M Gjomarkaj M Haahtela T Holgate ST Johnston SL Viruses and bacteria in acute asthma exacerbations–a GA^2^ LEN-DARE systematic review. *Allergy.* (2011) 66:458–68. 10.1111/j.1398-9995.2010.02505.x 21087215 PMC7159474

[29] Papadopoulos NG Christodoulou I Rohde G Agache I Almqvist C Bruno A Viruses and bacteria in acute asthma exacerbations–A GA^2^ LEN-DARE systematic review. *Allergy.* (2011) 66:458–68. 21087215 10.1111/j.1398-9995.2010.02505.x PMC7159474

[30] Turunen R Koistinen A Vuorinen T Arku B Söderlund-Venermo M Ruuskanen O The first wheezing episode: respiratory virus etiology, atopic characteristics, and illness severity. *Pediatr Allergy Immunol.* (2014) 25:796–803. 10.1111/pai.12318 25444257 PMC7167827

[31] Holt PG Mok D Panda D Renn L Fabozzi G deKlerk NH Developmental regulation of type 1 and type 3 interferon production and risk for infant infections and asthma development. *J Allergy Clin Immunol.* (2019) 143:1176–82.e5. 10.1016/j.jaci.2018.08.035 30217468

[32] Isaacs A Lindenmann J. Classics in oncology: Virus interference: I. the interferon. *CA Cancer J Clin.* (1988) 38:280–90. 10.3322/canjclin.38.5.280 2458172

[33] Sheppard P Kindsvogel W Xu W Henderson K Schlutsmeyer S Whitmore TE IL-28, IL-29 and their class II cytokine receptor IL-28R. *Nat Immunol.* (2003) 4:63–8. 10.1038/ni873 12469119

[34] Kotenko SV Gallagher G Baurin VV Lewis-Antes A Shen M Shah NK IFN-lambdas mediate antiviral protection through a distinct class II cytokine receptor complex. *Nat Immunol.* (2003) 4:69–77. 10.1038/ni875 12483210

[35] Kotenko SV Durbin JE. Contribution of type III interferons to antiviral immunity: location, location, location. *J Biol Chem.* (2017) 292:7295–303. 10.1074/jbc.R117.777102 28289095 PMC5418032

[36] Forero A Ozarkar S Li H Lee CH Hemann EA Nadjsombati MS Differential activation of the transcription Factor IRF1 underlies the distinct immune responses elicited by type I and type III interferons. *Immunity.* (2019) 51:451–64.e6. 10.1016/j.immuni.2019.07.007 31471108 PMC7447158

[37] Pervolaraki K Rastgou Talemi S Albrecht D Bormann F Bamford C Mendoza JL Differential induction of interferon stimulated genes between type I and type III interferons is independent of interferon receptor abundance. *PLoS Pathog.* (2018) 14:e1007420. 10.1371/journal.ppat.1007420 30485383 PMC6287881

[38] Klinkhammer J Schnepf D Ye L Schwaderlapp M Gad HH Hartmann R IFN-λ prevents influenza virus spread from the upper airways to the lungs and limits virus transmission. *eLife.* (2018) 7:e33354. 10.7554/eLife.33354 29651984 PMC5953542

[39] Lazear HM Schoggins JW Diamond MS. Shared and distinct functions of type I and type III interferons. *Immunity.* (2019) 50:907–23. 10.1016/j.immuni.2019.03.025 30995506 PMC6839410

[40] Galani IE Triantafyllia V Eleminiadou EE Koltsida O Stavropoulos A Manioudaki M Interferon-λ mediates non-redundant front-line antiviral protection against influenza virus infection without compromising host fitness. *Immunity.* (2017) 46:875–90.e6. 10.1016/j.immuni.2017.04.025 28514692

[41] Broggi A Tan Y Granucci F Zanoni I. IFN-λ suppresses intestinal inflammation by non-translational regulation of neutrophil function. *Nat Immunol.* (2017) 18:1084–93. 10.1038/ni.3821 28846084 PMC5701513

[42] Rich HE McCourt CC Zheng WQ McHugh KJ Robinson KM Wang J Interferon lambda inhibits bacterial uptake during influenza superinfection. *Infect Immun.* (2019) 87:e00114-19. 10.1128/IAI.00114-19 30804099 PMC6479047

[43] Planet PJ Parker D Cohen TS Smith H Leon JD Ryan C Lambda interferon restructures the nasal microbiome and increases susceptibility to staphylococcus aureus superinfection. *mBio.* (2016) 7:e01939-15. 10.1128/mBio.01939-15 26861017 PMC4752601

[44] Hendricks MR Lashua LP Fischer DK Flitter BA Eichinger KM Durbin JE Respiratory syncytial virus infection enhances *Pseudomonas Aeruginosa* biofilm growth through dysregulation of nutritional immunity. *Proc Natl Acad Sci USA.* (2016) 113:1642–7. 10.1073/pnas.1516979113 26729873 PMC4760822

[45] Goswami R Majumdar T Dhar J Chattopadhyay S Bandyopadhyay SK Verbovetskaya V Viral degradasome hijacks mitochondria to suppress innate immunity. *Cell Res.* (2013) 23:1025–42. 10.1038/cr.2013.98 23877405 PMC3731571

[46] García-Sastre A. Induction and evasion of type I interferon responses by influenza viruses. *Virus Res.* (2011) 162:12–8. 10.1016/j.virusres.2011.10.017 22027189 PMC3640439

[47] Bao X Liu T Shan Y Li K Garofalo RP Casola A. Human metapneumovirus glycoprotein G inhibits innate immune responses. *PLoS Pathog.* (2008) 4:e1000077. 10.1371/journal.ppat.1000077 18516301 PMC2386556

[48] Parisien JP Bamming D Komuro A Ramachandran A Rodriguez JJ Barber G A shared interface mediates paramyxovirus interference with antiviral RNA helicases MDA5 and LGP2. *J Virol.* (2009) 83:7252–60. 10.1128/JVI.00153-09 19403670 PMC2704796

[49] Eberle KC McGill JL Reinhardt TA Sacco RE. Parainfluenza virus 3 blocks antiviral mediators downstream of the interferon lambda receptor by modulating Stat1 phosphorylation. *J Virol.* (2015) 90:2948–58. 10.1128/JVI.02502-15 26719274 PMC4810625

[50] Wei F Jiang Z Sun H Pu J Sun Y Wang M Induction of PGRN by influenza virus inhibits the antiviral immune responses through downregulation of type I interferons signaling. *PLoS Pathog.* (2019) 15:e1008062. 10.1371/journal.ppat.1008062 31585000 PMC6795447

[51] Stone CA Miller EK. Understanding the association of human Rhinovirus with asthma. *Clin Vaccin Immunol.* (2015) 23:6–10. 10.1128/CVI.00414-15 26376925 PMC4711093

[52] Kalinowski A Galen BT Ueki IF Sun Y Mulenos A Osafo-Addo A Respiratory syncytial virus activates epidermal growth factor receptor to suppress interferon regulatory factor 1-dependent interferon-lambda and antiviral defense in airway epithelium. *Mucosal Immunol.* (2018) 11:958–67. 10.1038/mi.2017.120 29411775 PMC6431552

[53] Raundhal M Morse C Khare A Oriss TB Milosevic J Trudeau J High IFN-γ and low SLPI mark severe asthma in mice and humans. *J Clin Invest.* (2015) 125:3037–50. 10.1172/JCI80911 26121748 PMC4563754

[54] Gauthier M Chakraborty K Oriss TB Raundhal M Das S Chen J Severe asthma in humans and mouse model suggests a CXCL10 signature underlies corticosteroid-resistant Th1 bias. *JCI Insight.* (2017) 2:e94580. 10.1172/jci.insight.94580 28679952 PMC5499373

[55] Bullens DM Decraene A Dilissen E Meyts I De Boeck K Dupont LJ Type III IFN-lambda mRNA expression in sputum of adult and school-aged asthmatics. *Clin Exp Allergy.* (2008) 38:1459–67. 10.1111/j.1365-2222.2008.03045.x 18564328

[56] da Silva J Hilzendeger C Moermans C Schleich F Henket M Kebadze T Raised interferon-β, type 3 interferon and interferon-stimulated genes – evidence of innate immune activation in neutrophilic asthma. *Clin Exp Allergy.* (2017) 47:313–23. 10.1111/cea.12809 27622317

[57] Hastie AT Steele C Dunaway CW Moore WC Rector BM Ampleford E Complex association patterns for inflammatory mediators in induced sputum from subjects with asthma. *Clin Exp Allergy.* (2018) 48:787–97. 10.1111/cea.13129 29520864 PMC6319629

[58] Bhakta NR Christenson SA Nerella S Solberg OD Nguyen CP Choy DF IFN-stimulated gene expression, type 2 inflammation, and endoplasmic reticulum stress in asthma. *Am J Respir Crit Care Med.* (2018) 197:313–24. 10.1164/rccm.201706-1070OC 29064281 PMC5811952

[59] Huber JP Ramos HJ Gill MA Farrar JD. Cutting edge: type I IFN reverses human Th2 commitment and stability by suppressing GATA3. *J Immunol.* (2010) 185:813–7. 10.4049/jimmunol.1000469 20554961 PMC2927323

[60] Huber JP Gonzales-van Horn SR Roybal KT Gill MA Farrar JD. IFN-alpha suppresses GATA3 transcription from a distal exon and promotes H3K27 trimethylation of the CNS-1 enhancer in human Th2 cells. *J Immunol.* (2014) 192:5687–94. 10.4049/jimmunol.1301908 24813204 PMC4104489

[61] Shibuya H Hirohata S. Differential effects of IFN-alpha on the expression of various TH2 cytokines in human CD4+ T cells. *J Allergy Clin Immunol.* (2005) 116:205–12. 10.1016/j.jaci.2005.03.016 15990796

[62] Gonzales-van Horn SR Farrar JD. Interferon at the crossroads of allergy and viral infections. *J Leukoc Biol.* (2015) 98:185–94. 10.1189/jlb.3RU0315-099R 26026068 PMC4501675

[63] Pritchard AL White OJ Burel JG Upham JW. Innate interferons inhibit allergen and microbial specific T(H)2 responses. *Immunol Cell Biol.* (2012) 90:974–7. 10.1038/icb.2012.39 22825591

[64] Moschen AR Geiger S Krehan I Kaser A Tilg H. Interferon-alpha controls IL-17 expression in vitro and in vivo. *Immunobiology.* (2008) 213:779–87. 10.1016/j.imbio.2008.07.022 18926293

[65] Ramgolam VS Sha Y Jin J Zhang X Markovic-Plese S. IFN-beta inhibits human Th17 cell differentiation. *J Immunol.* (2009) 183:5418–27. 10.4049/jimmunol.0803227 19783688

[66] Wark PA Johnston SL Bucchieri F Powell R Puddicombe S Laza-Stanca V Asthmatic bronchial epithelial cells have a deficient innate immune response to infection with Rhinovirus. *J Exp Med.* (2005) 201:937–47. 10.1084/jem.20041901 15781584 PMC2213100

[67] Webb LM Lundie RJ Borger JG Brown SL Connor LM Cartwright AN Type I interferon is required for T helper (Th) 2 induction by dendritic cells. *EMBO J.* (2017) 36:2404–18. 10.15252/embj.201695345 28716804 PMC5556270

[68] Mattei F Bracci L Tough DF Belardelli F Schiavoni G. Type I IFN regulate DC turnover in vivo. *Eur J Immunol.* (2009) 39:1807–18. 10.1002/eji.200939233 19544312

[69] Rouzaut A Garasa S Teijeira A González I Martinez-Forero I Suarez N Dendritic cells adhere to and transmigrate across lymphatic endothelium in response to IFN-α. *Eur J Immunol.* (2010) 40:3054–63. 10.1002/eji.201040523 21061437

[70] Li Y Gao Q Yuan X Zhou M Peng X Liu X Adenovirus expressing IFN-λ1 (IL-29) attenuates allergic airway inflammation and airway hyperreactivity in experimental asthma. *Int Immunopharmacol.* (2014) 21:156–62. 10.1016/j.intimp.2014.04.022 24819718

[71] Won J Gil CH Jo A Kim HJ. Inhaled delivery of interferon-lambda restricts epithelial-derived Th2 inflammation in allergic asthma. *Cytokine.* (2019) 119:32–6. 10.1016/j.cyto.2019.02.010 30861490

[72] Koltsida O Hausding M Stavropoulos A Koch S Tzelepis G Ubel C IL-28A (IFN-λ2) modulates lung DC function to promote Th1 immune skewing and suppress allergic airway disease. *EMBO Mol Med.* (2011) 3:348–61. 10.1002/emmm.201100142 21538995 PMC3377081

[73] Jordan WJ Eskdale J Srinivas S Pekarek V Kelner D Rodia M Human interferon lambda-1 (IFN-lambda1/IL-29) modulates the Th1/Th2 Response. *Genes Immun.* (2007) 8:254–61. 10.1038/sj.gene.6364382 17361203

[74] Srinivas S Dai J Eskdale J Gallagher GE Megjugorac NJ Gallagher G. Interferon-lambda1 (interleukin-29) preferentially down-regulates interleukin-13 over other T helper type 2 cytokine responses in vitro. *Immunology.* (2008) 125:492–502. 10.1111/j.1365-2567.2008.02862.x 18547367 PMC2612545

[75] Johnston SL Pattemore PK Sanderson G Smith S Lampe F Josephs L Community study of role of viral infections in exacerbations of asthma in 9-11 year old children. *BMJ.* (1995) 310:1225–9. 10.1136/bmj.310.6989.1225 7767192 PMC2549614

[76] Henrickson KJ. Parainfluenza viruses. *Clin Microbiol Rev.* (2003) 16:242–64. 10.1128/cmr.16.2.242-264.2003 12692097 PMC153148

[77] Holtzman MJ Shornick LP Grayson MH Kim EY Tyner JW Patel AC “Hit-and-run” effects of paramyxoviruses as a basis for chronic respiratory disease. *Pediatr Infect Dis J.* (2004) 23 (11 Suppl.):S235–45. 10.1097/01.inf.0000144674.24802.c1 15577579

[78] Zheng XY Xu YJ Guan WJ Lin LF. Regional, age and respiratory-secretion-specific prevalence of respiratory viruses associated with asthma exacerbation: a literature review. *Arch Virol.* (2018) 163:845–53. 10.1007/s00705-017-3700-y 29327237 PMC7087223

[79] Garcia-Garcia ML Calvo C Ruiz S Pozo F Del Pozo V Remedios L Role of viral coinfections in asthma development. *PLoS One.* (2017) 12:e0189083. 10.1371/journal.pone.0189083 29206851 PMC5716580

[80] Baraldo S Contoli M Bazzan E Turato G Padovani A Marku B Deficient antiviral immune responses in childhood: distinct roles of atopy and asthma. *J Allergy Clin Immunol.* (2012) 130:1307–14. 10.1016/j.jaci.2012.08.005 22981791

[81] Contoli M Message SD Laza-Stanca V Edwards MR Wark PAB Bartlett NW Role of deficient type III interferon-λ production in asthma exacerbations. *Nat Med.* (2006) 12:1023–6. 10.1038/nm1462 16906156

[82] Zhu J Message SD Mallia P Kebadze T Contoli M Ward CK Bronchial mucosal IFN-α/β and pattern recognition receptor expression in patients with experimental rhinovirus-induced asthma exacerbations. *J Allergy Clin Immunol.* (2019) 143:114–25.e4. 10.1016/j.jaci.2018.04.003 29698627 PMC6320262

[83] Pritchard AL White OJ Burel JG Carroll ML Phipps S Upham JW. Asthma is associated with multiple alterations in anti-viral innate signalling pathways. *PLoS One.* (2014) 9:e106501. 10.1371/journal.pone.0106501 25203745 PMC4159236

[84] Sykes A Edwards MR Macintyre J del Rosario A Bakhsoliani E Trujillo-Torralbo MB Rhinovirus 16-induced IFN-α and IFN-β are deficient in bronchoalveolar lavage cells in asthmatic patients. *J Allergy Clin Immunol.* (2012) 129:1506–14.e6. 10.1016/j.jaci.2012.03.044 22657407

[85] Ravanetti L Dijkhuis A Dekker T Sabogal Pineros YS Ravi A Dierdorp BS IL-33 drives influenza-induced asthma exacerbations by halting innate and adaptive antiviral immunity. *J Allergy Clin Immunol.* (2019) 143:1355–70.e16. 10.1016/j.jaci.2018.08.051 30316823

[86] Edwards MR Regamey N Vareille M Kieninger E Gupta A Shoemark A Impaired innate interferon induction in severe therapy resistant atopic asthmatic children. *Mucosal Immunol.* (2013) 6:797–806. 10.1038/mi.2012.118 23212197 PMC3684776

[87] Simpson JL Carroll M Yang IA Reynolds PN Hodge S James AL Reduced antiviral interferon production in poorly controlled asthma is associated with neutrophilic inflammation and high-dose inhaled corticosteroids. *Chest.* (2016) 149:704–13. 10.1016/j.chest.2015.12.018 26836898

[88] Cerps SC Menzel M Mahmutovic Persson I Bjermer L Akbarshahi H Uller L. Interferon-β deficiency at asthma exacerbation promotes MLKL mediated necroptosis. *Sci Rep.* (2018) 8:4248. 10.1038/s41598-018-22557-6 29523863 PMC5844912

[89] Duerr CU Fritz JH. Regulation of group 2 innate lymphoid cells. *Cytokine.* (2016) 87:1–8. 10.1016/j.cyto.2016.01.018 27255596

[90] Hatchwell L Collison A Girkin J Parsons K Li J Zhang J Toll-like receptor 7 governs interferon and inflammatory responses to Rhinovirus and is suppressed by IL-5-induced lung Eosinophilia. *Thorax.* (2015) 70:854–61. 10.1136/thoraxjnl-2014-205465 26108570 PMC4552894

[91] Shikhagaie MM Andersson CK Mori M Kortekaas Krohn I Bergqvist A Dahl R Mapping of TLR5 and TLR7 in central and distal human airways and identification of reduced TLR expression in severe asthma. *Clin Exp Allergy.* (2014) 44:184–96. 10.1111/cea.12176 24447081

[92] Durrani SR Montville DJ Pratt AS Sahu S DeVries MK Rajamanickam V Innate immune responses to rhinovirus are reduced by the high-affinity IgE receptor in allergic asthmatic children. *J Allergy Clin Immunol.* (2012) 130:489–95. 10.1016/j.jaci.2012.05.023 22766097 PMC3437329

[93] Gill MA Bajwa G George TA Dong CC Dougherty II Jiang N Counterregulation between the FcepsilonRI pathway and antiviral responses in human plasmacytoid dendritic cells. *J Immunol.* (2010) 184:5999–6006. 10.4049/jimmunol.0901194 20410486 PMC4820019

[94] Teach SJ Gill MA Togias A Sorkness CA Arbes SJ Jr. Calatroni A Preseasonal treatment with either omalizumab or an inhaled corticosteroid boost to prevent fall asthma exacerbations. *J Allergy Clin Immunol.* (2015) 136:1476–85. 10.1016/j.jaci.2015.09.008 26518090 PMC4679705

[95] Kloepfer KM Olenec JP Lee WM Liu G Vrtis RF Roberg KA Increased H1N1 infection rate in children with asthma. *Am J Respir Crit Care Med.* (2012) 185:1275–9. 10.1164/rccm.201109-1635OC 22366048 PMC3381233

[96] Samarasinghe AE Woolard SN Boyd KL Hoselton SA Schuh JM McCullers JA. The immune profile associated with acute allergic asthma accelerates clearance of influenza virus. *Immunol Cell Biol.* (2014) 92:449–59. 10.1038/icb.2013.113 24469764 PMC4037497

[97] Nicholas B Dudley S Tariq K Howarth P Lunn K Pink S Susceptibility to influenza virus infection of bronchial biopsies in asthma. *J Allergy Clin Immunol.* (2017) 140:309–12.e4. 10.1016/j.jaci.2016.12.964 28259448

[98] Veerapandian R Snyder JD Samarasinghe AE. Influenza in asthmatics: for better or for worse? *Front Immunol.* (2018) 9:1843. 10.3389/fimmu.2018.01843 30147697 PMC6095982

[99] Jha A Dunning J Tunstall T Thwaites RS Hoang LT Kon OM Patterns of systemic and local inflammation in patients with asthma hospitalised with influenza. *Eur Respir J.* (2019) 54:1900949. 10.1183/13993003.00949-2019 31391224 PMC7612747

[100] McKenna JJ Bramley AM Skarbinski J Fry AM Finelli L Jain S. Asthma in patients hospitalized with pandemic influenza A(H1N1)pdm09 virus infection-United States, 2009. *BMC Infect Dis.* (2013) 13:57. 10.1186/1471-2334-13-57 23369034 PMC3585510

[101] Myles P Nguyen-Van-Tam JS Semple MG Brett SJ Bannister B Read RC Differences between asthmatics and nonasthmatics hospitalised with influenza a infection. *Eur Respir J.* (2013) 41:824–31. 10.1183/09031936.00015512 22903963 PMC3612580

[102] Ni YN Chen G Sun J Liang BM Liang ZA. The effect of corticosteroids on mortality of patients with influenza pneumonia: a systematic review and meta-analysis. *Crit Care.* (2019) 23:99. 10.1186/s13054-019-2395-8 PMC6437920 30917856

[103] Van Kerkhove MD Vandemaele KA Shinde V Jaramillo-Gutierrez G Koukounari A Donnelly CA Risk factors for severe outcomes following 2009 influenza A (H1N1) infection: a global pooled analysis. *PLoS Med.* (2011) 8:e1001053. 10.1371/journal.pmed.1001053 21750667 PMC3130021

[104] An S Jeon YJ Jo A Lim HJ Han YE Cho SW Initial influenza virus replication can be limited in allergic asthma through rapid induction of type III interferons in respiratory epithelium. *Front Immunol.* (2018) 9:986. 10.3389/fimmu.2018.00986 29867963 PMC5966536

[105] LeMessurier KS Rooney R Ghoneim HE Liu B Li K Smallwood HS Influenza A virus directly modulates mouse Eosinophil responses. *J Leukoc Biol.* (2020) 108:151–68. 10.1002/JLB.4MA0320-343R 32386457 PMC7859173

[106] Samarasinghe AE Melo RC Duan S LeMessurier KS Liedmann S Surman SL Eosinophils promote antiviral immunity in mice infected with influenza A virus. *J Immunol.* (2017) 198:3214–26. 10.4049/jimmunol.1600787 28283567 PMC5384374

[107] Sabogal Piñeros YS Bal SM Dijkhuis A Majoor CJ Dierdorp BS Dekker T Eosinophils capture viruses, a capacity that is defective in asthma. *Allergy.* (2019) 74:1898–909. 10.1111/all.13802 30934128 PMC6852198

[108] Matsuse H Tsuchida T Fukahori S Kawano T Tomari S Matsuo N Differential airway inflammatory responses in asthma exacerbations induced by respiratory syncytial virus and influenza virus A. *Int Arch Allergy Immunol.* (2013) 161:378–82. 10.1159/000348381 23689185

[109] Yokouchi Y Katsumori H Shirakawa S Fujiwara M Kashima K Kozawa R Protective effects of influenza A (H1N1) pandemic 2009 vaccination against the onset of influenza-like illness and asthma exacerbation in Japanese children. *J Asthma.* (2014) 51:825–31. 10.3109/02770903.2014.915567 24739075

[110] Kramarz P DeStefano F Gargiullo PM Davis RL Chen RT Mullooly JP Does influenza vaccination exacerbate asthma? Analysis of a large cohort of children with asthma. vaccine safety datalink team. *Arch Fam Med.* (2000) 9:617–23. 10.1001/archfami.9.7.617 10910309

[111] Kramarz P DeStefano F Gargiullo PM Davis RL Chen RT Mullooly JP Influenza vaccination in children with asthma in health maintenance organizations. vaccine safety datalink team. *Vaccine.* (2000) 18:2288–94. 10.1016/s0264-410x(99)00551-4 10717349

[112] Ravanetti L Dijkhuis A Sabogal Pineros YS Bal SM Dierdorp BS Dekker T An early innate response underlies severe influenza-induced exacerbations of asthma in a novel steroid-insensitive and anti-IL-5-responsive mouse model. *Allergy.* (2017) 72:737–53. 10.1111/all.13057 27696462

[113] Li BWS de Bruijn MJW Lukkes M van Nimwegen M Bergen IM KleinJan A T cells and ILC2s are major effector cells in influenza-induced exacerbation of allergic airway inflammation in mice. *Eur J Immunol.* (2019) 49:144–56. 10.1002/eji.201747421 29762870 PMC6585726

[114] Wilson NG Hernandez-Leyva A Kau AL. The ABCs of wheeze: asthma and bacterial communities. *PLoS Pathog.* (2019) 15:e1007645. 10.1371/journal.ppat.1007645 31022286 PMC6483249

[115] Sintobin I Siroux V Holtappels G Pison C Nadif R Bousquet J Sensitisation to Staphylococcal Enterotoxins and asthma severity: a longitudinal study in the EGEA cohort. *Eur Respir J.* (2019) 54:1900198. 10.1183/13993003.00198-2019 31285304

[116] Talbot TR Hartert TV Mitchel E Halasa NB Arbogast PG Poehling KA Asthma as a risk factor for invasive pneumococcal disease. *N Engl J Med.* (2005) 352:2082–90. 10.1056/NEJMoa044113 15901861

[117] Damjanovic D Khera A Medina MF Ennis J Turner JD Gauldie J Type 1 interferon gene transfer enhances host defense against pulmonary *Streptococcus pneumoniae* infection via activating innate leukocytes. *Mol Ther Methods Clin Dev.* (2014) 1:5. 10.1038/mtm.2014.5 26015944 PMC4378291

[118] Bisgaard H Hermansen MN Buchvald F Loland L Halkjaer LB Bønnelykke K Childhood asthma after bacterial colonization of the airway in neonates. *N Engl J Med.* (2007) 357:1487–95. 10.1056/NEJMoa052632 17928596

[119] Biesbroek G Tsivtsivadze E Sanders EA Montijn R Veenhoven RH Keijser BJ Early respiratory microbiota composition determines bacterial succession patterns and respiratory health in children. *Am J Respir Crit Care Med.* (2014) 190:1283–92. 10.1164/rccm.201407-1240OC 25329446

[120] King PT Sharma R. The lung immune response to nontypeable *Haemophilus Influenzae* (Lung Immunity to NTHi). *J Immunol Res.* (2015) 2015:706376. 10.1155/2015/706376 26114124 PMC4465770

[121] Alnahas S Hagner S Raifer H Kilic A Gasteiger G Mutters R IL-17 and TNF-α are key mediators of *Moraxella catarrhalis* triggered exacerbation of allergic airway inflammation. *Front Immunol.* (2017) 8:1562. 10.3389/fimmu.2017.01562 29184554 PMC5694487

[122] Heinrich A Haarmann H Zahradnik S Frenzel K Schreiber F Klassert TE *Moraxella catarrhalis* decreases antiviral innate immune responses by down-regulation of TLR3 via inhibition of p53 in human bronchial epithelial cells. *FASEB J.* (2016) 30:2426–34. 10.1096/fj.201500172R 26979086

[123] Roberts S Salmon SL Steiner DJ Williams CM Metzger DW Furuya Y. Allergic airway disease prevents lethal synergy of influenza A virus-*Streptococcus pneumoniae* coinfection. *mBio.* (2019) 10:e01335-19. 10.1128/mBio.01335-19 31266877 PMC6606812

[124] Halwani R Al-Muhsen S Al-Jahdali H Hamid Q. Role of transforming growth factor-β in airway remodeling in asthma. *Am J Respir Cell Mol Biol.* (2011) 44:127–33. 10.1165/rcmb.2010-0027TR 20525803

[125] LeMessurier KS Iverson AR Chang TC Palipane M Vogel P Rosch JW Allergic inflammation alters the lung microbiome and hinders synergistic Co-infection with H1N1 influenza virus and *Streptococcus pneumoniae* in C57BL/6 Mice. *Sci Rep.* (2019) 9:19360. 10.1038/s41598-019-55712-8 31852944 PMC6920369

[126] Goh KJ Yii ACA Lapperre TS Chan AK Chew FT Chotirmall SH Sensitization to *Aspergillus* species is associated with frequent exacerbations in severe asthma. *J Asthma Allergy.* (2017) 10:131–40. 10.2147/JAA.S130459 28461762 PMC5407445

[127] Takazono T Sheppard DC. Aspergillus in chronic lung disease: modeling what goes on in the airways. *Med Mycol.* (2017) 55:39–47. 10.1093/mmy/myw117 27838644

[128] Espinosa V Dutta O McElrath C Du P Chang YJ Cicciarelli B Type III interferon is a critical regulator of innate antifungal immunity. *Sci Immunol.* (2017) 2:eaan5357. 10.1126/sciimmunol.aan5357 28986419 PMC5880030

[129] Schnepf D Staeheli P. License to kill: IFN-λ regulates antifungal activity of neutrophils. *Sci Immunol.* (2017) 2:eaa9614. 10.1126/sciimmunol.aap9614 29150440

[130] McKeever T Harrison TW Hubbard R Shaw D. Inhaled corticosteroids and the risk of pneumonia in people with asthma: a case-control study. *Chest.* (2013) 144:1788–94. 10.1378/chest.13-0871 23990003

[131] Singanayagam A Glanville N Girkin JL Ching YM Marcellini A Porter JD Corticosteroid suppression of antiviral immunity increases bacterial loads and mucus production in COPD exacerbations. *Nat Commun.* (2018) 9:2229. 10.1038/s41467-018-04574-1 29884817 PMC5993715

[132] Numasaki M Tagawa M Iwata F Suzuki T Nakamura A Okada M IL-28 elicits antitumor responses against murine fibrosarcoma. *J Immunol.* (2007) 178:5086–98. 10.4049/jimmunol.178.8.5086 17404291

[133] Luo Q Liu Y Liu S Yin Y Xu B Cao J. Interleukin 28 is a potential therapeutic target for sepsis. *Clin Immunol.* (2019) 205:29–34. 10.1016/j.clim.2019.05.012 31121287

[134] Tak PP. IFN-beta in rheumatoid arthritis. *Front Biosci.* (2004) 9:3242–7. 10.2741/1475 15353352

[135] Liu X Diedrichs-Möhring M Wildner G. The role of IFN-alpha in experimental and clinical uveitis. *Ocul Immunol Inflamm.* (2019) 27:23–33. 10.1080/09273948.2017.1298822 28375033

[136] Li Y Hua S. Mechanisms of pathogenesis in allergic asthma: role of interleukin-23. *Respirology.* (2014) 19:663–9. 10.1111/resp.12299 24779686

[137] Gratzl S Palca A Schmitz M Simon HU. Treatment with IFN-alpha in corticosteroid-unresponsive asthma. *J Allergy Clin Immunol.* (2000) 105:1035–6. 10.1067/mai.2000.105317 10808188

[138] Simon HU Seelbach H Ehmann R Schmitz M. Clinical and immunological effects of low-dose IFN-alpha treatment in patients with corticosteroid-resistant asthma. *Allergy.* (2003) 58:1250–5. 10.1046/j.1398-9995.2003.00424.x 14616099

[139] Kroegel C Bergmann N Heider C Moeser A Happe J Schlenker Y Interferon-alpha as treatment option in severe persistent uncontrolled bronchial asthma: an open label study. *Pneumologie* (2009) 63:307–13. 10.1055/s-0029-1214738 19517357

[140] Maeda Y Musoh K Shichijo M Tanaka H Nagai H. Interferon-beta prevents antigen-induced bronchial inflammation and airway hyperreactivity in mice. *Pharmacology.* (1997) 55:32–43. 10.1159/000139510 9309799

[141] Djukanović R Harrison T Johnston SL Gabbay F Wark P Thomson NC The effect of inhaled IFN-β on worsening of asthma symptoms caused by viral infections. a randomized trial. *Am J Respir Crit Care Med.* (2014) 190:145–54. 10.1164/rccm.201312-2235OC 24937476 PMC4226052

[142] Duvall MG Barnig C Cernadas M Ricklefs I Krishnamoorthy N Grossman NL Natural killer cell-mediated inflammation resolution is disabled in severe asthma. *Sci Immunol.* (2017) 2:eaam5446. 10.1126/sciimmunol.aam5446 28783702 PMC5561743

[143] Oriss TB Raundhal M Morse C Huff RE Das S Hannum R IRF5 distinguishes severe asthma in humans and drives Th1 phenotype and airway hyperreactivity in mice. *JCI Insight.* (2017) 2:e91019. 10.1172/jci.insight.91019 28515358 PMC5436536

